
JOURNAL INFORMATION
==============================
NLM Title Abbreviation: J Diabetes Investig
Journal ID: J Diabetes Investig
NLM Journal ID: 101520702
Journal ID: JDI

Journal of Diabetes Investigation

ISSN: 2040-1116
EISSN: 2040-1124
Publisher: Wiley

ARTICLE INFORMATION
==============================
PMCID: PMC11474629
DOI: 10.1111/jdi.14198
Article ID: JDI14198
Article version: 1
Subjects: Issue Information, Issue Information

Issue Information

Electronic publication date: 2024 Mar 30
Publication date: 2024 Apr
Volume: 15
Issue: 4
First page: 391
Last page: 392
Copyright: © 2024 The Authors. Journal of Diabetes Investigation published by Asian Association for the Study of Diabetes (AASD) and John Wiley & Sons Australia, Ltd
License: This is an open access article under the terms of the http://creativecommons.org/licenses/by/4.0/ License, which permits use, distribution and reproduction in any medium, provided the original work is properly cited.
License URL: https://creativecommons.org/licenses/by/4.0/

Figure count: 0
Table count: 0
Page count: 5

source-schema-version-number: 2.0
cover-date: April 2024
details-of-publishers-convertor: Converter:WILEY_ML3GV2_TO_JATSPMC version:6.4.9 mode:remove_FC converted:15.10.2024

==============================

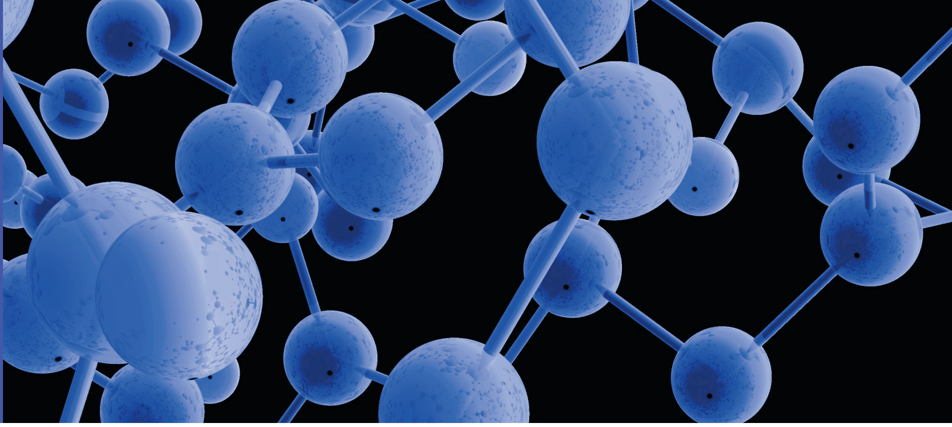

### JDI

Volume 15 | Issue 4
April 2024

**Journal of Diabetes Investigation**
Official Journal of the Asian Association for the Study of Diabetes

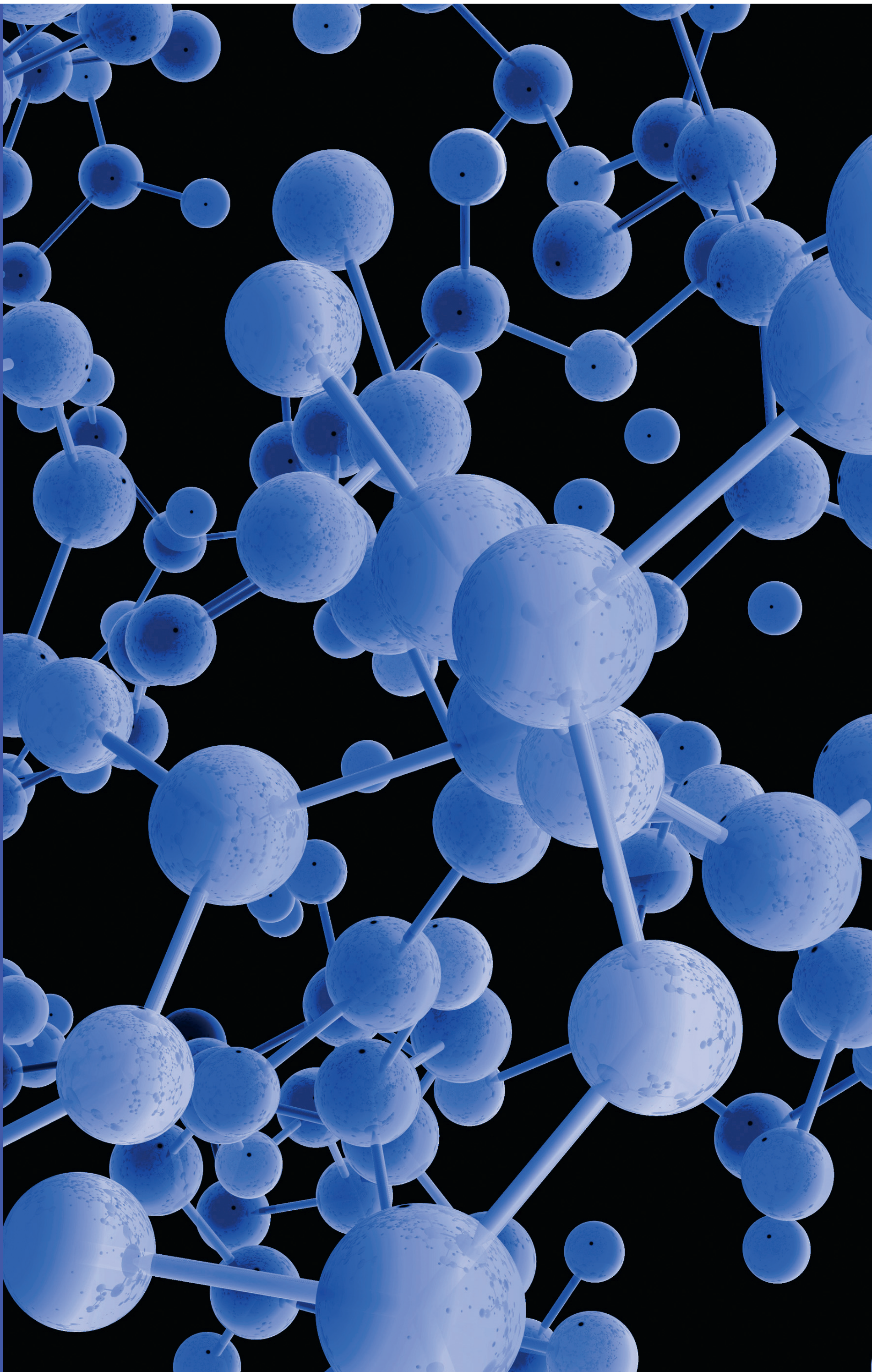

Aspirin use in
diabetes and CKD

THE ASIAN ASSOCIATION FOR THE STUDY OF DIABETES
WILEY

### JDI Journal of Diabetes Investigation

Official Journal of the Asian Association for the Study of Diabetes

Open access

**Editor-in-Chief**

Nigishi Hotta, *Japan Organization of Occupational Health and Safety, Chubu Rosai Hospital, Nagoya, Japan*

**Co-Editor**

Domenico Accili, *Columbia University, New York, USA*

**Expert Editor**

Bo Ahren, *Lund, Sweden*
Young Min Cho, *Seoul, Korea*
Sean Dinneen, *Galway, Ireland*
Marc Gilbert, *Montpelier, France*
Limei Liu, *Shanghai, China*
Ronald Ma, *Hong Kong, China*
Louis Philipson, *Chicago, USA*
Wayne H-H Sheu, *Taichung, Taiwan*
Bernard Thorens, *Lausanne, Switzerland*
Daisuke Yabe, *Gifu, Japan*

**Editorial Board**

Eiichi Araki, *Kumamoto, Japan*
Xiaoping Chen, *Beijing, China*
Lee-Ming Chuang, *Taipei, Taiwan*
Masakazu Haneda, *Asahikawa, Japan*
Larry Low-Tone Ho, *Taipei, Taiwan*
Tianpei Hong, *Beijing, China*
Akihisa Imagawa, *Osaka, Japan*
Nobuya Inagaki, *Kyoto, Japan*
Linong Ji, *Beijing, China*
Weiping Jia, *Shanghai, China*
Yi-Der Jiang, *Taipei, Taiwan*
Takashi Kadowaki, *Tokyo, Japan*
Kohei Kaku, *Kurashiki, Japan*
Keizo Kanasaki, *Izumo, Japan*
Doo-Man Kim, *Seoul, Korea*
Karen Lam, *Hong Kong, China*
Hong Kyu Lee, *Gyeonggi-do, Korea*
In-Kyu Lee, *Daegu, Korea*
Moon-Kyu Lee, *Seoul, Korea*
Jiro Nakamura, *Nagakute, Japan*
Kishio Nanjo, *Wakayama, Japan*
Wataru Ogawa, *Kobe, Japan*
Yutaka Seino, *Osaka, Japan*
Shyi-Jang Shin, *Kaohsiung, Taiwan*
Hirohito Sone, *Niigata, Japan*
Kathryn Tan, *Hong Kong, China*
Yukio Tanizawa, *Ube, Japan*
Kohjiro Ueki, *Tokyo, Japan*
Jun Wada, *Okayama, Japan*
Hirotaka Watada, *Tokyo, Japan*
Jianping Weng, *Guangzhou, China*
Soroku Yagihashi, *Hirosaki, Japan*
Hiroshi Yamamoto, *Kanazawa, Japan*
Toshimasa Yamauchi, *Tokyo, Japan*
Wenying Yang, *Beijing, China*
Kazuki Yasuda, *Tokyo, Japan*
Koutaro Yokote, *Chiba, Japan*
Kun-Ho Yoon, *Seoul, Korea*
Dalong Zhu, *Nanjing, China*

**International Editorial Board**

Michael A. Brownlee, *New York, USA*
Subrata Chakrabarti, *Ontario, Canada*
Mark E. Cooper, *Prahran, Australia*
Philippe Froguel, *London, UK*
Wilfred Fujimoto, *Hawaii, USA*
Michael S. German, *San Francisco, USA*
Andrew Hattersley, *Exeter, UK*
Jean-Claude Henquin, *Brussels, Belgium*
Jens Juul Holst, *Copenhagen, Denmark*
Frank B. Hu, *Boston, USA*
C. Ronald Kahn, *Boston, USA*
George L. King, *Boston, USA*
Rayaz A. Malik, *Manchester, UK*
Michael Mauer, *Minneapolis, USA*
Carl Erik Mogensen, *Aarhus, Denmark*
Steven E. Shoelson, *Boston, USA*
Jay S. Skyler, *Miami, USA*
Paul Zimmet, *Melbourne, Australia*

**Assistant Editorial Board**

Tien-Jyun Chang, *Taipei, Taiwan*
Yoshiyuki Hamamoto, *Osaka, Japan*
Norio Harada, *Kyoto, Japan*
Yi-Jen Hung, *Taipei, Taiwan*
Chi-Min Hwu, *Taipei, Taiwan*
Hideki Kamiya, *Aichi, Japan*
Dae Jung Kim, *Suwon, Korea*
Seung-Hwan Lee, *Seoul, Korea*
Hung-Yuan Li, *Taipei, Taiwan*
Takashi Matsuzaka, *Tsukuba, Japan*
Keiko Naruse, *Aichi, Japan*
Takeshi Nishikawa, *Kumamoto, Japan*
Eung-Jung Rhee, *Seoul, Korea*
Yusuke Seino, *Aichi, Japan*
Hironori Waki, *Tokyo, Japan*

**Aims and Scope:** *Journal of Diabetes Investigation* is your core diabetes journal from Asia; the official journal of the Asian Association for the Study of Diabetes (AASD). The journal publishes original research, country reports, commentaries, reviews, mini-reviews, case reports, letters, as well as editorials and news. The journal embraces clinical and experimental research in diabetes and related areas. This includes aspects of prevention, treatment, as well as molecular aspects and pathophysiology. Translational research focused on the exchange of ideas between clinicians and researchers is also welcome.

**Cover:** Insulin molecule © Pasieka/Science Photo Library

This journal is available online at Wiley Online Library. Visit wileyonlinelibrary.com to search the articles and register for table of contents and e-mail alerts.

**For submission instructions, and all other information visit wileyonlinelibrary.com/journal/jdi**

JDI.JEB.Apr24

**Open Access and Copyright**
All articles published by *Journal of Diabetes Investigation* are fully open access: immediately freely available to read, download and share. All *Journal of Diabetes Investigation* articles accepted from 24 September 2013 are published under the terms of the Creative Commons Attribution 4.0 Unported (CC BY 4.0) license, which permits use, distribution and reproduction in any medium, provided the original work is properly cited. Articles accepted before this date were published under the agreement as stated in the final article. Copyright on any *Journal of Diabetes Investigation* article accepted after 24 September 2013, the date on which *Journal of Diabetes Investigation* became an open access journal, is retained by the author(s). Authors grant to *Journal of Diabetes Investigation* and Wiley a license to publish the article and for Wiley to identify itself as the original publisher. Authors also grant any third party the right to use the article freely as long as its integrity is maintained and its original authors, citation details and publisher are identified. Further information about open access licenses and copyright can be found at: https://authorservices.wiley.com/author-resources/Journal-Authors/licensing/licensing-info-faqs.html#4. Information on the licenses used by Journal of Diabetes Investigation can be found at: wileyonlinelibrary.com/journal/jdi

**Disclaimer**
The Publisher, the Asian Association for the Study of Diabetes, and Editors cannot be held responsible for any errors in or any consequences arising from the use of information contained in this journal. The views and opinions expressed do not necessarily reflect those of the Publisher, the Asian Association for the Study of Diabetes or Editors, neither does the publication of advertisements constitute any endorsement by the Publisher, the Asian Association for the Study of Diabetes, Editors, or Authors, of the products advertised.

Wiley Open Access articles posted to repositories or websites are without warranty from Wiley of any kind, either express or implied, including, but not limited to, warranties of merchantability, fitness for a particular purpose, or non-infringement. To the fullest extent permitted by law Wiley disclaims all liability for any loss or damage arising out of, or in connection, with the use of or inability to use the content.

JDI.PI.Apr24

VOLUME 15 – ISSUE 4 – APRIL 2024

### Journal of Diabetes Investigation

Open access

### Reviews

**Resistant starch and type 2 diabetes mellitus: Clinical perspective**
Min Kyung Kim, Juri Park, and Doo-Man Kim 395

**Changing landscape of diabetes in Asia – What are the unmet needs?**
Andrea OY Luk 402

**Perspectives on genetic studies of type 2 diabetes from the genome-wide association studies era to precision medicine**
Minako Imamura and Shiro Maeda 410

### Commentaries

**Advancements and challenges in pancreatic islet transplantation: Insights from the Collaborative Islet Transplant Registry**
Eun Young Lee and Kun-Ho Yoon 423

**Deciphering the causal tapestry between cardiorespiratory fitness and type 2 diabetes mellitus**
Fu-Shun Ko, Yun-Kai Yeh, Fu-Shun Yen, and Chii-Min Hwu 426

### Original Articles

### Basic Science and Research

**Epac2 activation mediates glucagon-induced glucogenesis in primary rat hepatocytes**
Yusuke Shiozaki-Takagi, Nobuaki Ozaki, and Yukiyasu Toyoda 429

### Clinical Science and Care

**Short-term recovery of insulin secretion in response to a meal is associated with future glycemic control in type 2 diabetes patients**
Asako Enkaku, Daisuke Chujo, Miki Kamigishi, Shinya Inagawa, Shinnosuke Matsukoshi, Waka Sakai, Akiko Takikawa, Shiho Fujisaka, and Kazuyuki Tobe 437

### Clinical Trial

**Comparative effects of fixed-dose mitiglinide/voglibose combination and glimepiride on vascular endothelial function and glycemic variability in patients with type 2 diabetes: A randomized controlled trial**
Kenichi Tanaka, Yosuke Okada, Saeko Umezu, Ryoma Hashimoto, Yukiko Tomoyose, Rina Tateyama, Yuri Hori, Momo Saito, Akemi Tokutsu, Satomi Sonoda, Fumi Uemura, Akira Kurozumi, and Yoshiya Tanaka 449

### Original Articles

**Low-dose aspirin for prevention of cardiovascular mortality in patients with type 2 diabetes and chronic kidney disease: A real-world nationwide cohort study**
Yi-Cheng Lin, Bi-Li Chen, Wan-Ting Chen, Li-Nien Chien, and Chun-Yao Huang 459

**Development and validation of a prediction model for self-reported hypoglycemia risk in patients with type 2 diabetes: A longitudinal cohort study**
Hongmei Xu, Hangqing Yu, Zhengnan Cheng, Chun Mu, Di Bao, Xiaohui Li, and Qiuling Xing 468

**Inverted U-shaped associations between serum uric acid and fasting - plasma glucose level in non-diabetic, pre-diabetic, and diabetic adults: A population-based study in China**
Hongjuan Shi, Yining Liu, Danyu Yang, Peifeng Liang, Chen Chen, Hong Luan, and Chao Shi **483**

**Estimated small dense low-density lipoprotein cholesterol and nonalcoholic fatty liver disease in nonobese populations**
Hangkai Huang, Jiarong Xie, Linxiao Hou, Min Miao, Lei Xu, and Chengfu Xu **491**

**Comparing the screening methods for gestational diabetes mellitus before and during the COVID-19 pandemic: A systematic review**
Xingge Sun, Clare McKeaveney, Helen Noble, Hannah O'Hara, and Oliver Perra **500**

### Letter to the Editor

**Comment on the article titled "Association of the triglyceride-glucose index with subclinical left ventricular dysfunction in type 2 diabetes mellitus patients: A retrospective cross-sectional study"**
Mehmet Ilkin Naharci **517**
